# Receptors Involved in Mental Disorders and the Use of Clozapine, Chlorpromazine, Olanzapine, and Aripiprazole to Treat Mental Disorders

**DOI:** 10.3390/ph16040603

**Published:** 2023-04-17

**Authors:** Ronald Mlambo, Jia Liu, Qian Wang, Songwen Tan, Chuanpin Chen

**Affiliations:** Department of Pharmacy, Xiangya School of Pharmaceutical Sciences, Central South University, Changsha 410013, China

**Keywords:** 5-HT, clozapine, chlorpromazine, receptors, antipsychotic

## Abstract

Mental illnesses are a global health challenge, and effective medicines are needed to treat these conditions. Psychotropic drugs are commonly prescribed to manage mental disorders, such as schizophrenia, but unfortunately, they can cause significant and undesirable side effects, such as myocarditis, erectile dysfunction, and obesity. Furthermore, some schizophrenic patients may not respond to psychotropic drugs, a condition called schizophrenia-treatment resistance. Fortunately, clozapine is a promising option for patients who exhibit treatment resistance. Unlike chlorpromazine, scientists have found that clozapine has fewer neurological side effects. Additionally, olanzapine and aripiprazole are well-known for their moderating effects on psychosis and are widely used in clinical practice. To further maximize drug efficacy, it is critical to deeply understand the receptors or signaling pathways central to the nervous system, such as serotonin, histamine, trace amines, dopamine, and G-protein coupled receptors. This article provides an overview of the receptors mentioned above, as well as the antipsychotics that interact with them, such as olanzapine, aripiprazole, clozapine, and chlorpromazine. Additionally, this article discusses the general pharmacology of these medications.

## 1. Introduction

People and animals experience events that trigger stress, depression, anxiety, and sometimes mental disorders. Treatment of mental disorders is daunting because most of the medications used pose serious side effects. The adverse effects include disturbed sleeping patterns and a suicidal mentality [1]. These adverse effects may manifest as disturbed sleeping patterns and suicidal tendencies, making such drugs less than ideal, despite their therapeutic effects [2]. Psychotropic drugs commonly used for schizophrenia have been found to have severe side effects such as myocarditis, erectile dysfunction, and obesity. Moreover, some patients with schizophrenia may not respond to psychotropic drugs, a condition known as schizophrenia-treatment resistance. There is a pressing need to develop efficient drugs that have minimal to no side effects. Clozapine (CZP) has been identified as a more favorable option for such patients, as it is associated with fewer neurological side effects than chlorpromazine (CPZ). Furthermore, the medications olanzapine (OZP) and aripiprazole (ARP) are commonly prescribed for their ability to moderate various symptoms [3,4,5,6,7].

The dopaminergic system plays a crucial role in motor performance, cognitive function, and emotional behavior. Dysfunctions in dopamine (DA) transmission has been associated with the development of mental illnesses, such as depression and schizophrenia. Research has shown abnormal DA levels in the post-mortem brains of patients with schizophrenia, along with increased DA metabolites and receptor levels in the ventral striatum. There is a notion that overstimulation of D2 receptors could be accountable for schizophrenia-positive symptoms. Scientists further found out that levodopa, a DA precursor, and amphetamine, a DA-releasing agent, worsened the symptoms of schizophrenia [1,4,5,8,9,10,11,12,13,14,15,16]. Neuroimaging studies have revealed that schizophrenic patients display an escalated release of DA in the ventral striatum after amphetamine induction, indicating heightened sensitivity of their dopaminergic system when compared with a control group. The manifestation of negative symptoms correlates with diminished dopaminergic activity of the mesocortical pathway. Similar results were obtained when stimulation of D1, D3, and D4 receptors in the prefrontal cortex was reduced, leading to the appearance of negative symptoms [17,18,19,20].

The current leading theory regarding the pathophysiology of depression posits that the disorder arises from a disruption in monoaminergic transmission. This pertains specifically to three key monoamines: 5-Hydroxytryptamine (5-HT), noradrenaline (NE), and DA. This hypothesis is grounded in the observation that antidepressant medications work to increase the availability of at least one of these neurotransmitters. It is believed that impaired monoaminergic transmission may result from monoamine depletion, altered synthesis and regulation of monoamine activity (including reuptake by specific transporters), or changes in the expression of monoamine receptors [17,21,22,23,24,25]. Functional connectivity of monoaminergic neurons involves direct or indirect interconnections between NE, 5-HT, and DA neurons. These connections are mediated through various receptors, which act upon both heteroreceptors and autoreceptors. The impact of 5-HT systems on DA and NE neurotransmission were observed to be complicated through 5-HT2A and 5-HT2C receptor-mediated mode of action. The NE system also has complicated positive and negative impacts on the 5-HT neurotransmission. Similarly, the NE system has positive and negative impacts on 5-HT neurotransmission, which are mediated via α1 and α2 adrenergic receptors, respectively. Therefore, the multimodal effect on central monoamine neurotransmission impact on the reuptake transporters and the different monoamine auto/heteroreceptors appears to improve the efficacy of the resistant depression treatment [26,27,28].

The aim of this article is to examine four antipsychotic medications—OZP, ARP, CZP, and CPZ—and their therapeutic effects on specific receptors. Additionally, the article delves into the impact of these antipsychotics on various receptors, such as serotonin, histamine, trace amines, DA, and G-protein coupled receptors (GPCRs). Table 1 shows some antipsychotic drugs and the receptors they target.

## 2. Central Nervous System Receptors and Mental Disorders

Mounting evidence suggests that various mental disorders are linked to receptor signaling pathways. Key receptors implicated in developing numerous mental ailments include DA, 5-HT, and histamine, among others. The following sections will explore the relationship between these receptors and mental disorders. Antipsychotic medications bind to and/or act on several different receptors, resulting in a variety of mechanisms being employed. These mechanisms involve the interaction of serotonin, histamine, trace amines, DA, and GPCPs with atypical antipsychotics (APYs) [38].

### 2.1. Histamine Receptors’ Involvement in Mental Disorders

The APYs, such as quetiapine (antagonist), OZP (agonist), and CZP (agonist), interact and bind to histamine-1 (H1) receptors in a way that is comparable to their interactions with other receptors, such as α2-adrenoceptors, D2, and 5-HT2A/2C receptors. Scientists concluded that the central nervous system (CNS) medications that interact with histamine receptors are responsible for adverse effects on the immune system. Nonetheless, the fact that central histamine is associated with emotions, sleep, cognition, and memory means that the therapeutic potential of histamine-binding medicines attracted attention. Therefore, studies in vivo between monoamines and histamine interactions were conducted [39,40]. As such, studies have been conducted to investigate the interactions between monoamines and histamine in vivo, with commonly studied monoamines, including serotonin, DA, and norepinephrine. Microdialysis and electrophysiological experiments were conducted to investigate the effects of histamine-3/4 (H3/4) receptor partial agonism on histamine levels in the brain. It was found that the histamine-3 (H3) selective antagonist thioperamide increased extracellular levels of DA, serotonin, and norepinephrine in the hypothalamus and prefrontal cortex (PFC) while also potentiating DA-firing activity. However, the drug did not increase the levels of norepinephrine or serotonin neurons. These findings suggest that H1 agonists and H3 antagonists may offer significant therapeutic benefits in treating psychotic and mood disorders, as they can enhance monoamine transmission in the brain [38].

### 2.2. Trace Amines’ Receptors and Neurotransmitters Associated with Mental Illness

Scientists have discovered that trace amines are naturally occurring chemical molecules closely resembling biogenic neurotransmitters such as norepinephrine, DA, and serotonin. The levels of trace amines in the CNS are typically very deficient, hence their name. These critical chemicals include tryptamine, p-tryptamine, p-octapamine, β-phenylethylamine, and their metabolites [41].

History clearly reveals that since the discovery of trace amine-associated receptors (TAAR), scientists have been attracted to GPCPs with the intention of manufacturing medicines that treat psychotic ailments. The trace amine-associated receptor 1 (TAAR1) was the most-studied receptor. It was observed that TAAR1 is activated by several endogenous chemical compounds, such as serotonin, DA, norepinephrine, and monoamine neurotransmitters, as well as some of these chemical metabolites. All TAAR genes are clustered in a genomic area spanning 108 kb in chromosome 6q23, a susceptibility locus for schizophrenia and other mental disorders. Scientists tried to spot the gene loci on the susceptibility locus to see if regions are associated with mental disorders. Nonetheless, the information acquired was not sufficient to have a clear significant inference [41].

According to Dedic et al., the five studied selective TAAR1 agonists, namely RO5263397, RO5203648, RO5256390, RO5256390, and RO5073012, showed water-promoting, antidepressant-like, antipsychotic, and anti-addictive properties [41]. These effects were thought to be exerted through serotonergic, glutamatergic, and dopaminergic circuits. One of the prominent TAAR1 agonists, ulotaront, succeeded in the phase 3 trials. The mode of action of the drug is not fully understood. However, experimental findings in vivo and in vitro revealed that ulotaront combines complete agonism at TAAR1 with a partial agonism at the serotonin-1D receptor and mild activity at serotonin-7 receptors.

### 2.3. Mechanisms That Involve DA Receptors in Mental Diseases

DA is a catecholamine neurotransmitter that plays a key role in regulating both the CNS and the peripheral nervous system (PNS). Signaling involving DA transmission is mediated by a group of GPCRs, divided into two categories: D1-like and D2-like receptors. D1-like receptors, including D1 receptors (D1R) and D5 receptors (D5R), activate stimulatory G proteins (Gs), while the second group of D2-like receptors (D2R, D3R, and D4R) activate inhibitory G proteins (Gi/o). Howbeit, D1R and D2R dominate the CNS, particularly in the prefrontal cortex and the basal ganglia. Scientists report that neuropsychiatric ailments, such as schizophrenia, autism, and Parkinson’s disease, are associated with D1R- and D2R-signaling pathways. Therefore, several ligands (medications) were developed that target the two receptors to treat CNS disorders and keep the dopaminergic system constant [42,43,44,45].

**Figure 1:**
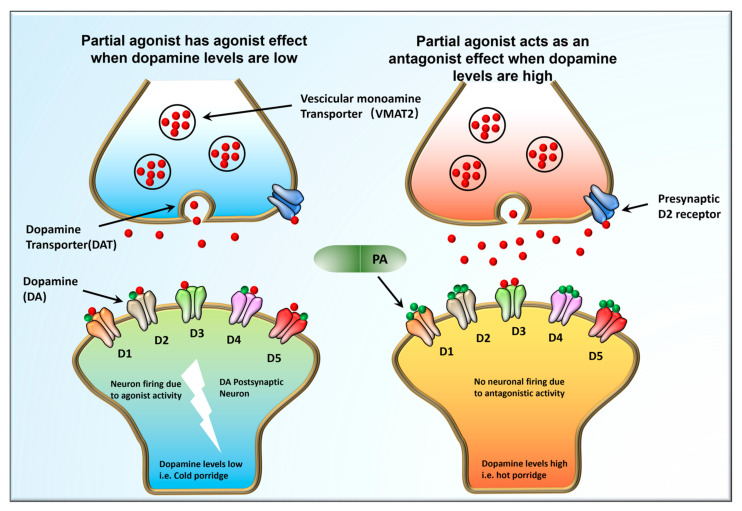
The Goldilocks effect. Dopaminergic signaling pathways in between neurons during impulse firing. Partial DA receptor inhibitors can adjust DA levels in individuals depending on the DA levels the body demands at that particular given time. ARP is an example of the partial D2 receptor that is used to mitigate both positive and negative symptoms of schizophrenia.

It is documented that biochemical pathways that involve the transmission of DA influence the onset of psychoses. Therefore, any antagonistic pathway is antipsychotic and pro-convulsive. Antipsychotic drugs exert their therapeutic effect by blocking the subcortical D2 receptors [46]. Scientists hypothesize that changes in D1 and D2 receptor signaling may activate neuronal cell death cascades through the protein kinase A (PKA)/extracellular-regulated kinase (ERK) and mammalian target rapamycin (mTOR) pathways [19,47]. Partial inhibitors of the DA receptor are highly effective in regulating DA levels based on the specific needs of an individual at any given time. ARP is an example of the partial D2 receptor that is used to mitigate both positive and negative symptoms of schizophrenia (Figure 1).

According to Zhang et al.’s experiments, it was found that despite belonging to the same DA receptor family, D1R and D2R differ in their phylogenetic ‘neighbors’, with D1R being closer to β-adrenergic receptors (βADRs) that couple with the Gs. Although ligands such as epinephrine can activate βADRs with less potency, the two DA receptors’ agonists, SKF81297 and SKF83959, used in Zhang and colleagues’ research share a similar chemical structure but have varying binding affinities and power to induce Gs. Their findings revealed that, of the two SKF chemicals’ ability to stimulate Gs-cyclo monophosphate (Gs-cAMP) pathways, only SKF81297 was observed to stimulate β-arrestin recruitment, despite both chemicals’ potential in this regard [28].

### 2.4. GPKs (G-Protein Coupled Receptor Kinases) and GPCRs Involvement in Psychoses Etiology

**Figure 2:**
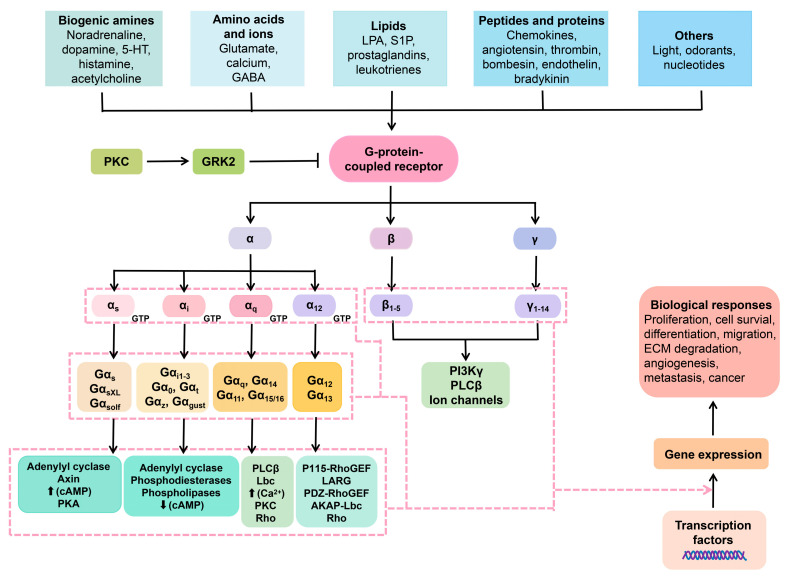
Membrane GPCRs and the intracellular cascade pathways in relation to neuropharmacy. Ligands interact with the extracellular GPCRs. The GPCRs are harnessed to heterotrimeric protein units, namely the α, β, and γ. Once the GCPRs perceive the ligands (ions, lipids, proteins, and so on), the heterotrimeric protein disengage in a manner that the α subunit exchanges GDP for GTP, hence activation. Once that occurs, a number of signal transduction pathways can take place.

GCPRs play a crucial role in transmitting information from a wide range of ligands and neurotransmitters, including but not limited to glutamate, monoamines, lipids, and GABA (gamma-aminobutyric acid) [48]. GPKs, in turn, phosphorylate GPCR proteins, which is a crucial regulatory mechanism responsible for receptor turnover/desensitization [49]. The interaction between GPKs and GPCRs is known to regulate a variety of physiological processes, as shown in Figure 2. Interestingly, recent research indicates that GPKs also regulate non-GPCR targets through phosphorylation-dependent or -independent mechanisms and play a role in biological activities, such as cell proliferation, growth, and immune modulation. Mutations occurring in GPKs can potentially initiate the development of psychiatric and neurological disorders [50].

One study found that GPK 3-deficient mice displayed functional and biochemical traits that closely resembled those observed in patients with psychotic syndromes. The proteomic analysis also revealed abnormally high levels of peptides associated with bipolar disorder and schizophrenia [51]. These findings suggest that a malfunction in dopaminergic transmission may play a key pathophysiological role in both disorders. Additionally, the study found that striatal dopaminergic activity in response to amphetamine was elevated, a common observation in imaging studies of patients with schizophrenia.

### 2.5. β-Arrestins and Psychoses

When β-arrestins 1 and 2 were discovered, they were named for their capability to sterically perturb the G protein coupling of agonist-activated seven-transmembrane receptors (7TMRs), which ultimately resulted in receptor desensitization [52]. Arrestins effectively hinder GPCR-related signaling pathways through the phosphorylation of GPCR cytosolic tails. However, recent findings have revealed that β-arrestins not only block GPCR signaling pathways but are also capable of independently activating signaling cascades [53]. Moreover, β-arrestins serve as multiprotein scaffolds and bring elements of different specific signaling pathways closer to each other. The regulation of the β-arrestins has been demonstrated in many signaling molecules, such as the mitogen-activated protein kinases (MAPK), ERK (Extracellular signal-regulated kinase), JNK (c-Jun N-terminal kinase), p38, Akt, PI3 kinase, and RhoA. In a noteworthy experiment, it was observed that β-arrestin-dependent ERK signaling plays a crucial role in reducing anxiety-like and conditioned fear-related behaviors in mice. This then implies that malfunctions in the β-arrestins involved in the ERK signaling could lead to anxiety and conditioned fear-related behaviors [54].

## 3. Psychotropic Drugs Usage

**Figure 3:**
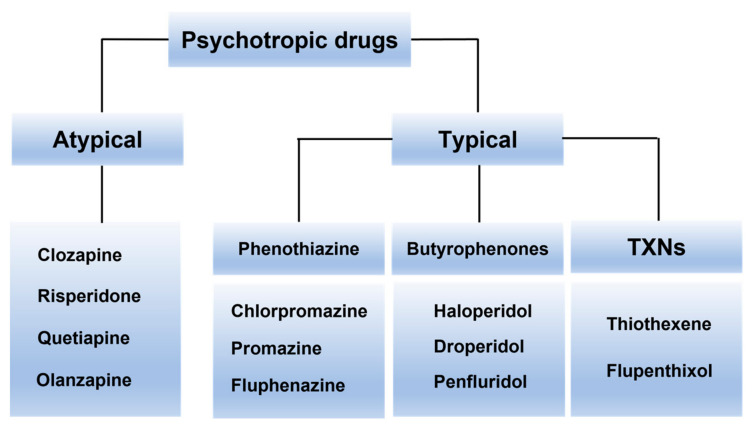
Classification of antipsychotic medicines and their corresponding examples. TXNs are thioxanthenes. There is also another category known as other heterocyclic psychotropic drugs.

The World Health Organisation (WHO) defines psychotropic medications as agents that exert their effects on the CNS, thereby altering mood, behavior, and cognition. These medications are classified into categories, such as sedatives, antipsychotics, hypnotics, mood stabilizers, and antidepressants. Figure 3 illustrates the classification and examples of antipsychotic drugs. The major classes of psychotropic medications that are frequently used are antidepressants and antipsychotics [42]. This is due to the advancement in diagnosis in the field of psychiatry [30]. China is among the leading countries in terms of the utilization of psychotropic medications. According to a national population-based study on psychotropic consumption in China, antidepressants (48.4%) are the most frequently prescribed medication, followed by sedatives (31.3%) and antipsychotics (15.6%). These figures are based on data gathered from 2018 to 2021 [55].

### 3.1. APYs as Adjuncts to Antidepressants Treat Schizophrenia and Many Other Mental Problems via Serotonin Receptors

**Figure 4:**
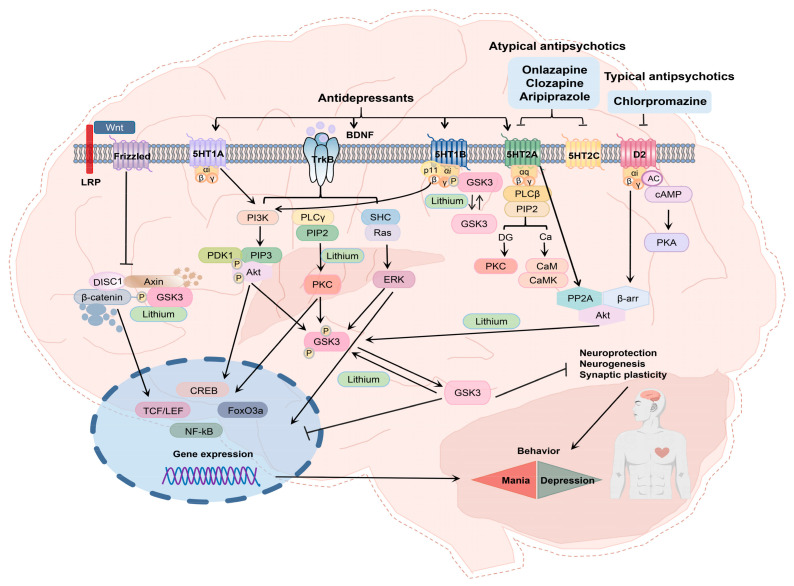
The effects of atypical psychotropic drugs (CZP, OZP, and ARP), typical antipsychotics (CPZ), and antidepressants on quenching mania and depression. Lithium is also a common therapy for mania and depression. Lithium inhibits the enzyme glycogen synthase kinase 3 (GSK3) and then aids the phosphorylation process of the GSK3 at the N-terminal serine residue. In addition, lithium inhibits inositol phosphatases hence aborting phosphatidylinositol signaling. Antidepressants exert their effect via serotonin receptors, and the neurotrophic receptor activity is also facilitated. Atypical psychotropics CZP and OZP block both D2 and 5-HT2A receptors. Stimulation of these receptors results in either activation or inhibition of protein kinase C (PKC), Akt (protein kinase B), and ERK (extracellular signal-regulated kinase) via different signaling pathways. The GSK3 is phosphorylated by the Akt and PKC at the N-terminal serine residue and GSK3 phosphorylation by the ERK at the C-terminal serine results in the inactivation of the GSK3. TMX inhibits the PKC activity and thus regulation of mood. AC (adenyl cyclase), Ca (calcium), cAMP (cyclic adenosine monophosphate), CaM (calmodulin), CaMK (calmodulin-dependent protein kinase), CREB (cAMP-responsive element binding protein), DG (diacylglycerol), DISC1 (disrupted in schizophrenia 1), FoxO (forkhead ‘O’ transcription factor), IP3 (inositol triphosphate), LRP5/6 (low-density lipoprotein receptor-related protein-5/6), β-arr (β-arrestin), TrkB (tyrosine kinase-B), PP2 (protein phosphatase-2), PP1 (protein phosphatase-1), PLC (phospholipase C), PKA (protein kinase A), PIP3 (phosphatidylinositol (3,4,5)-trisphosphate), PIP2 (phosphatidylinositol (4,5)-bisphosphate), PI3K (phosphoinositide 3-kinase), PDK1 (phosphoinositide-dependent protein kinase 1), NF-kB (Nuclear factor-kappa beta), and MEK (mitogen-activated protein kinase).

The history of APYs dates back to the 1990s. They have since been found to be highly effective in treating the cognitive and negative symptoms of schizophrenia. In addition to treating schizophrenia, APYs have also been used to address affective disorders, either as standalone treatments or in combination with antidepressants. The atypical psychotics work by modulating norepinephrine, 5-HT, and/or neurotransmission of histamine. The mode of action of APYs is multimodal and is deemed to be the reason behind the beneficial effects in treating mood disorders and anxiety. Moreover, novel medications, such as adenosine, histamine, and trace amine-associated receptor ligands, also function through the multimodal stimulatory mechanism of central serotonin, norepinephrine, and/or histamine neurotransmission [56]. The combination of antipsychotic and antidepressant medications has been suggested to work through the same mechanism of central monoamine neurotransmission, leading to a potential combined effect [38,57,58]. While APYs are often effective as monotherapies in treating bipolar patients, they are not as effective in treating unipolar depression. However, certain APYs, such as risperidone, ARP, quetiapine, OZP, and asenapine, have been shown to effectively treat bipolar depression, with the exception of asenapine, which can also treat general anxiety. In the case of unipolar depression, APYs are often administered in combination with antidepressants, such as serotonin and norepinephrine reuptake inhibitors (SNRIs) or selective serotonin reuptake inhibitors (SSRIs) (Figure 4). If depression does not respond to treatment, adjunct therapies have been found to be highly effective. The combination of antidepressants with APYs, such as risperidone, ARP, and brexpiprazole, has proven to be an excellent adjunct treatment, effectively overcoming resistant depression [38].

The latest research indicates that serotonin receptors can be regulated to control the transmission of serotonin, providing a potential treatment option for epilepsy. The receptors are categorized into seven families (5-HT1–7). Amongst these receptors, 5-HT1A is the most studied. The 5-HT1A agonists have demonstrated antidepressant, cognition-enhancing, anti-epileptic, neuroprotective, and anxiolytic effects, making them a popular choice in treating depression and anxiety [59]. Buspirone and tandospirone are the two most widely used agonists. In animal models, inhibition of the 5-HT1A receptors has shown to be anticonvulsive. Notably, 5-HT1A antagonists have proven to reduce spike-wave discharge in rats, further supporting the role of serotonin receptors in treating epilepsy [60,61,62,63].

### 3.2. CPZ

The use of first-generation antipsychotics (FGAMs) in treating schizophrenia has been a topic of debate within the medical community. Some argue that these medications are not thoroughly studied in randomized control trials (RCTs) to assess their efficacy and safety. As a result, FGAMs should not be the initial recommended treatment for schizophrenia when better alternatives are available. However, it should be noted that certain FGAMs have shown promising pharmacological properties that could potentially make them safer and more effective, pending additional RCTs to support this hypothesis [64].

CPZ, classified as a phenothiazine antipsychotic drug, was first synthesized in 1950 and was considered a major breakthrough of its time. The medication improved and helped schizophrenic patients to lead almost everyday lives. Psychopharmacological studies revealed that CPZ might cause severe side effects, such as sleep disorders, hypotension, weight gain, Parkinson’s-like, and extrapyramidal dysfunctions. Therefore, the drug has been replaced by better medications, particularly second-generation antipsychotics. Nonetheless, CPZ remains a powerful antipsychotic agent for research purposes to understand antipsychotic behavior and dopaminergic signaling pathways. The drug CPZ is known to readily cross the blood–brain barrier (BBB) and interact with a wide range of receptors, leading to its versatility but also the potential for serious side effects. CPZ exerts its effects via an antagonism mode of actions, specifically by blocking M1 and M2 acetylcholine (Ach) receptors, DA receptors (D1–D4), histamine (H1) receptors, α1 and α2 adrenergic receptors, and serotonin (5-HT2, 5-HT6, and 5-HT7) receptors. The reduction of DA activity as such explains the antipsychotic properties of CPZ [65].

CPZ is a lipophilic drug that is slow to clear from the body. Scientists say that CPZ is deemed a low-potency FGAM that mainly causes non-neurological adverse effects. Blockage of muscarinic receptors results in blurred vision, constipation, dry mouth, urine retention, and dizziness. Sedation is a result of H1 receptor blockage. In adults, the risk of angle-closure glaucoma is reported. Additionally, CPZ has been found to cause hyperprolactinemia, which is thought to be due to its D2 receptor-blocking effects. Once hyperprolactinemia is triggered, libido goes down both in men and women. Male individuals may experience erectile dysfunction, gynecomastia, and galactorrhea. Less frequently, priapism in males has been reported. Women with hyperprolactinemia may experience irregular menstrual patterns, galactorrhea, oligomenorrhea, and amenorrhea [65].

Patients that receive CPZ via intravenous or intramuscular routes may suffer from headaches and hypotension. Individuals who use the drug for quite a long may lead to lens opacity and corneal deposits. Moreover, CPZ can hinder bile flow in the liver, resulting in cholestatic jaundice and hepatotoxicity, which can cause ALT levels to rise. As a result, liver function tests are necessary for therapeutic drug monitoring (TDM). Once prominent levels of ALT are detected, a patient must refrain from CPZ treatment [66].

CPZ is primarily metabolized in the kidneys and the liver, with the CYP2D6 protein governing the main metabolic pathway. Furthermore, other isozymes, such as CYP1A2 and CYP3A4, are also involved in the metabolism of CPZ. To date, approximately 10–12 CPZ metabolites have been identified. After hydroxylation at positions 3 and 7 of the phenothiazine CPZ nucleus, the N-dimethylaminopropyl methyl group is demethylated, yielding a product that is further metabolized to N-oxide. It is reported that in urine, 20% of CPZ and its derivative metabolites are eliminated from the system as single compounds, unconjugated. The rest are conjugated, and this portion mainly contains O-glucuronides and trace amounts of mono- and di-hydroxyl CPZ metabolites. About 37% of the CPZ administered dose is excreted in urine [67].

### 3.3. The Interaction of CPZ with Anti-COVIDS and Other Drugs

Delirium, schizophrenia, and anxiety, among other mental disorders, can be a challenge when patients under CPZ treatment are also taking COVID-19 medicines. The risks of principal interactions between antipsychotics and COVID-19 medicines include QT prolongation, CYP 450 enzyme interactions, and TdP (Torsade de Pointes). Anti-COVID-19 drugs, such as baricinitib, anakinra, and remdesivir, can be concomitantly administrated with CPZ without the risk of drug-to-drug interactions. The antiviral drug favipiravir needs caution when co-administered with CPZ. Scientists assert that tocilizumab is rather safe when co-administered with CPZ [68]. Table 2 shows the effects of co-administering CPZ with other drugs. 

### 3.4. CZP

Second-generation antipsychotics (SGAMs) are highly effective medications that are preferred for treating schizophrenic and psychotic disorders [70]. The SGAMs are of particular interest in the United States of America because of their anti-suicidal effects. These medications have been shown to reduce suicide cases, making them a valuable treatment option. One SGAM that stands out is CZP [71]. 

CZP is one of the unique antipsychotics because of its ability to treat resistant schizophrenia. Unlike many other antipsychotics, CZP has few severe neurological side effects and is known for improving negative symptoms somehow. In addition, it is effective in treating aggressive behavior [72]. CZP belongs to the tricyclic dibenzodiazepine antipsychotic medication category and requires therapeutic drug monitoring (TDM) due to the risk of agranulocytosis [73].

CZP is an antagonist to 5-HT and DA receptors. The affinity for the D4 receptor is higher than that of the D2 receptors, which accounts for its ability to mitigate adverse effects. CZP also partially binds to 5-HT1A receptors, which has been found to reduce negative side effects, such as extrapyramidal symptoms. The drug also binds to muscarinic receptors, including M1, M2, M3, and M5. One of the metabolites, norclozapine (NCPZ), has been shown to bind to M1 and M4 receptors. Moreover, CZP acts as an alpha-1-adrenergic and histamine antagonist [74].

Different CYP 450 enzymes are involved in the metabolism of CZP, including CYP2D6, 2C19, 3A4, and 1A2. Scientists recorded the involvement of CYP2D6 and C9 enzymes in metabolizing CZP to NCPZ [74].

According to De Berardis et al., the chances of developing agranulocytosis in patients under a CZP regimen are slim, that is, 0.01. Consequently, scientists say that the occurrence of agranulocytosis is independent of CZP dosing. Myocarditis is also a potential adverse effect of CZP use, occurring in approximately 3% of patients. CZP increases insulin resistance, and, as a result, patients gain weight and suffer from type 2 diabetic ketoacidosis and hyperlipidemia. Other side effects include sexual dysfunction, urinary retention, constipation, pulmonary embolism, seizures, and excessive salivation [74].

Generally, the bioavailability of CZP is typically around 60–70% when taken orally, owing to the liver’s first-pass effect. However, food does not seem to have any impact on the absorption of CPZ. Blood concentration peaks at approximately 160 min after oral ingestion, with a steady-state elimination half-life of almost 14 h. Furthermore, the distribution volume and plasma clearance are 1.6–7 L/kg and 8.7–53.3 L/h, respectively. It is reported that 95% of CPZ is bound to plasma proteins, mainly the α-1-glycoprotein [75]. 

It is documented that CPZ and NPCZ predict clinical outcomes. Therefore, regular therapeutic drug monitoring for these two is necessary to avoid adverse effects while ensuring drug safety. Although there is debate surrounding the exact therapeutic window for CPZ, most research suggests that a range of 350–600 ng/mL is associated with good clinical outcomes. However, it is essential to note that CPZ concentrations above 1000 ng/mL can increase the risk of seizures. The therapeutic range for NCPZ, the CPZ metabolite, is lower than that of the parent drug, ranging from 50–90% of the CPZ range. In elderly patients or when control is achieved, the CPZ lower limit concentration is 200 ng/mL [76].

### 3.5. ARP

**Figure 5:**
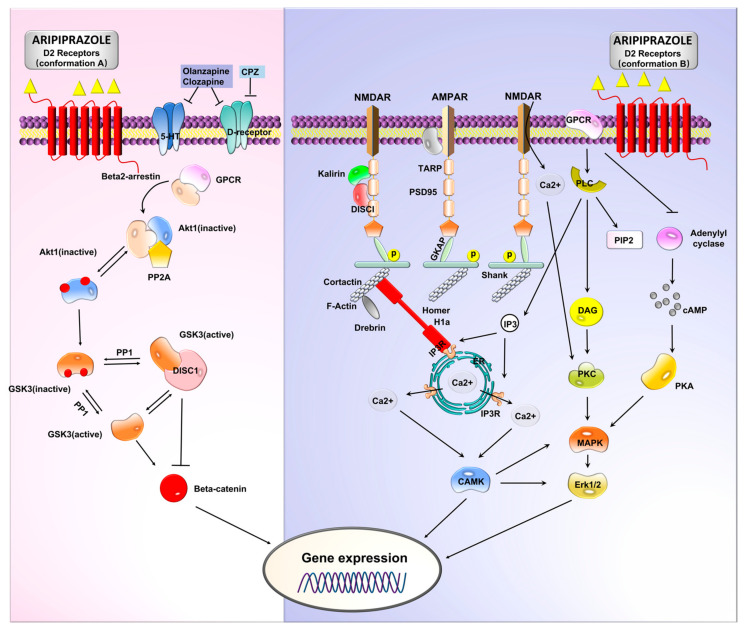
ARP postsynaptic functional selectivity. ARP causes what is referred to as functional multiplicity at D2 receptors’ postsynaptic downstream pathways. One mode of action of functional selectivity might be the preferential binding to different D2 receptor conformations, which have been demonstrated to activate differential transduction pathways according to the neuronal subtypes in which they are expressed. Postsynaptic scaffolding/adaptors and effectors might be affected in a different manner by each D2 receptor conformation-related pathway selectively activated by ARP. PSD-95 (postsynaptic density protein-95 kD), TARP (transmembrane AMPA receptors regulating protein or stargazing), MAPKs (mitogen-activated protein kinases), nMDAR (N-methyl-D-aspartate glutamate receptor), PDE4 (phosphodiesterase 4), GKAP (guanylate kinase-associated protein), and AMPAR (α-amino-3-hydroxy-5-methyl-4-isoxazolepropionic acid glutamate receptor).

The invention of the first D2 receptor agonist antipsychotic medicine paved the way for synthesizing new schizophrenia drugs. The introduction of the ARP significantly reduced the side effects that were associated with previous medications, such as extrapyramidal symptoms that change in severity depending on the receptor blockade level. Scientists report that the antagonistic effect is more robust in first-generation antipsychotic medicines, such as haloperidol, and weaker in second-generation ones, such as CZP or quetiapine. The ARP exerts its agonist effect by acting upon D2, D3, and serotonin 5-HT1A receptors, while its antagonist effect is exerted via 5-HT2A receptors. This ‘dual-effect’ characteristic of ARP brings about allowance in regulating positive, negative, and behavioral schizophrenic symptoms with minimum metabolic and extrapyramidal adverse effects. Partial agonist impact on the presynaptic D2 receptors is known to improve psychosis symptoms, but its partial agonism could also exacerbate the psychosis symptoms. However, the drug’s effect on 5-HT1A and 5-HT2A receptors creates a balance-like system, thereby stabilizing the DA–serotonin system. In addition, the medicine also exhibits moderate affinity for other receptors, such as α1, α2, and H1 receptors, and has been shown to have only marginal affinity for muscarinic receptors (Figure 5) [8,77].

ARP is commonly metabolized in the liver by CYP2D6 and CYP3A4. The metabolism involves three major biotransformation pathways: dehydrogenation, hydroxylation, and N-dealkylation. ARP is a prodrug converted to its active form, dehydro-ARP (D-ARP), after dehydrogenation. D-ARP is responsible for approximately 40% of the parent drug’s AUC in plasma at a steady state. The ARP’s pharmacokinetics is linear and dose-dependent within the range of 5–30 mg. Surprisingly, both ARP and its dehydrogenation metabolite show similar pharmacological characteristics. According to neuropsychopharmacology consensus guidelines for therapeutic drug monitoring, the ARP therapeutic window falls within the range of 10 to 350 μg/L whereas the active moiety, which is a combination of the parent drug and its dehydrogenation-metabolite, falls within the scope of 150–500 μg/L [23,78,79].

The desire for every pharmaceutical company is to develop a drug with a therapeutic range with the best clinical efficacy and minimal adverse effects. However, the notion is ideal, and achieving the goal is daunting because the evidence for inter-individual variability of drug plasma concentrations is piling up. The broad spectrum of pharmacokinetic variability for medicines metabolized by the CYP2D6 is primarily attributed to genetic polymorphism. Scientists have observed that the CYP2D6 gene is highly polymorphic, with over 100 allelic variants. The nomenclature of the isoforms places the variants into four different phenotypes, namely poor metabolizers (PMs), intermediate metabolizers (IMs), normal metabolizers (NMs), and ultra-rapid metabolizers (UMs). Due to genetic variation, the CYP2D6 PMs carry two non-functional (recessive) alleles, resulting in a lack of enzyme activity. Consequently, PMs show elevated plasma concentrations and a long half-life (t_1/2_) (146 h vs. 75 h) for ARP. Thus, it is essential to adjust doses based on the broad CYP-mediated metabolism and high inter-individual variability of ARP, as has been recommended for other psychotropic medications [22,43,63].

### 3.6. OZP

OZP, a member of the atypical psychotropic medicines, acts upon multiple receptors to exert its therapeutic effects. OZP exerts its antipsychotic effect by blocking D2 receptors, serotonin 5-HT2A receptors, and α1-adrenergic receptors, reducing neurotransmitter release and activity. It also possesses significant anticholinergic activity, can stimulate 5-HT1A receptors (agonistic effect), inhibit D4 receptors (antagonistic effect), and block H1 receptors (antagonistic effect). OZP shows anti-inflammatory properties, improves cognition, enhances sleep regulation, promotes neural regeneration, and can alleviate depression and emotional dysregulation. According to the U.S. Food and Drug Administration (FDA), OZP is indicated for the treatment of mainly two mental disorders, namely schizophrenia and bipolar I disorder. For children aged 13 years and above, a single dose of OZP monotherapy is effective. In case of depressive episodes that are in concert with bipolar I disorder, OZP is administered in combination with fluoxetine for children of age 10 years and above. Moreover, the medicine is administered off-label to children for several indications, such as eating, tic, attention deficit/hyperactivity, delirium, autism spectrum, and pervasive developmental disorders. In other words, OZP exhibits great versatility. The relationship between specific pharmacokinetic thresholds, such as pre-dose plasma concentrations and therapeutic efficacy for OZP, remains vague. Nonetheless, a few studies have shed light on the pharmacokinetics of OZP in children, particularly those who are under the age of 10 [11].

The oral bioavailability of OZP is as high as 60–65%, and it is distributed throughout the body’s tissues and organs via the gastrointestinal and liver pathways. It is metabolized in the liver through the CYP1A2 and CYP2D6 pathways, resulting in multiple metabolites. However, the principal metabolite which can affect OZP efficacy is N-demethylolanzapine, which has a lower conformational activity level than the original drug. It is mainly excreted through urine and feces, and the half-life of the drug is approximately 33 h, while the half-life of the metabolite N-demethylolanzapine is comparatively longer. OZP is highly bound to serum proteins, approximately 93–99%. It passes through the blood–brain barrier and is distributed throughout the body’s tissues and organs. OZP carries a risk of drug interactions and adverse reactions [80]. OZP not only affects the metabolism and absorption of other drugs but also is affected by other drugs, leading to enhanced effects or increased side effects. For example, co-administration with strong liver enzyme inducers (such as carbamazepine) can reduce the effectiveness of OZP, while co-administration with strong antifungal agents (such as ketoconazole) can increase OZP concentration. Co-administration with proton pump inhibitors, such as cimetidine, may reduce OZP’s bioavailability. In addition, OZP has many other clinical drug interactions that require careful use and monitoring. At the same time, OZP also has serious adverse reactions, such as arrhythmia, leukopenia, pancreatitis, diabetes, etc. The common adverse side effects reported in patients prescribed with OZP are drowsiness, weight gain, elevated prolactin serum, extrapyramidal reactions, tardive dyskinesia, and metabolic disorders, amongst others. The most severe adverse reaction is diabetes or hyperlipidemia which is caused by abnormal metabolism. Therefore, it is necessary to use OZP cautiously and monitor its response in pregnant and lactating women, patients with liver dysfunction, elderly patients, and patients with suicidal tendencies, alcoholism, and drug addiction [45]. 

According to the Arbeitsgemeinschaft für Neuropsychopharmakologie und Pharmakopsychiatri (AGNP) guidelines, the therapeutic window for OZP is described as 20 to 80 ng/mL. OZP is absorbed orally and is mostly metabolized into 10-N, 4′-N-glucuronides, and 4′-N-desmethyl-OZP by the hepatic CYP1A2. OZP is converted to OZP-N-oxide by the flavin-containing monooxygenase 3. It is reported that the drug concentration in the plasma reaches its steady state and peak concentration following 7 days and 6 h, respectively. Approximately 93% of OZP is bound to plasma proteins, with the CYP2D6 pathway metabolizing this percentage to produce 2-hydroxymethyl OZP. It is reported that serum OZP concentrations are affected by factors such as age, smoking habits, co-medications, and gender. One of the meta-analyses showed that OZP dose-related concentrations in patients with smoking behavior were significantly lower than in non-smokers [76]. Table 3 shows OZP, CPZ, CZP, and ARP mode of action and description.

## 4. Knowledge Gap

There remains a significant knowledge gap surrounding the use of antipsychotic drugs and their long-term effects. Despite this, it is common practice for patients to remain on medication for life, even after they have recovered from acute episodes. This can result in undesired medication effects, such as weight gain, which can impact both the length and quality of patients’ lives [81]. These observations were made from randomized and double-blind controlled studies. In addition, antipsychotics are not disease-modifying. This is deemed the most significant problem. Furthermore, clinical data show that the current schizophrenic treatment regimen effectively reduces positive symptoms but not harmful and cognitive symptoms. Therefore, there is still room for research to address the above problems, among others [82].

## 5. Conclusions

Further research on CNS receptors is essential to fully understand the mechanisms of antipsychotic drugs, as there is a significant need to address mental disorders worldwide, particularly schizophrenia. Despite being commonly used as a first-line treatment, these drugs often come with adverse and potentially fatal side effects. This is especially concerning, given that many patients with mental illnesses are prescribed antipsychotic medications for extended periods. It is crucial to prioritize research in this area to better understand and mitigate these risks and to ensure the safety and efficacy of these treatments in the long term.

## Figures and Tables

**Table 1:** Some antipsychotic drugs and the receptors they target.

Drug	Target Receptor (s)	Description	Reference/Link
Risperidone	D2, H1, α1 and α2, and 5-HT2A.	Mechanism of action not fully understood. Current studies focus more on how the drug blocks D2 and 5-HT2A receptors, making it an antagonist.	[29]
Quetiapine	Has affinity for D2, 5-HT2A, H1, α1, and 5-HT1A receptors	It is a second-generation antipsychotic drug. Acts as an antagonist for 5HT2A, D2, α1, and H1 receptors. It is also a 5HT1A partial agonist.	[30]
Promazine	Has an affinity for D1, D2, D4, 5-HT2A, 5-HT2C, H1, M1, M2, M3, M4, M5, and α1 receptors.	It is an antagonist for all the listed receptors.	[31]
Fluphenazine	Fluphenazine exerts its actions by blocking postsynaptic D2 receptors in the limbic and cortical systems and the basal ganglia.	A D2 antagonist that prevents the actions of DA, thereby reducing the hallucinations and delusions that are associated with schizophrenia.	[32]
Haloperidol	A first-generation antipsychotic that exerts a strong antagonism effect on D2 receptors.	Highly effective for the management of the “positive” symptoms of schizophrenia, including hallucinations, hearing voices, aggression/hostility, disorganized speech, and psychomotor agitation.	[33]
Droperidol	A D2 and α1 antagonist.	Droperidol is a butyrophenone derivative and a DA antagonist used to prevent and treat postoperative nausea and vomiting.	[34]
Penfluridol	Penfluridol inhibits the binding of DA-to-DA receptors with a Ki of 1.6 μM. It exerts its antipsychotic activity by blocking the DA projections in the limbic system and in the mesocortical area.	First-generation diphenylbutylpiperidine antipsychotic drug.	[35]
Thioperamide	An H3 antagonist.	It is used for the treatment of psychiatric disorders and cognitive disorders.	[36]
Promazine	Has an affinity for D1, D2, D4, 5-HT2A, 5-HT2C, H1, M1, M2, M3, M4, M5, and α1 receptors.	It is an antagonist for all the listed receptors.	[37]

**Table 2:** The effects of co-administering CPZ with other drugs [69].

Drug	Effects
Abacavir	High serum levels of abacavir due to a decreased rate of elimination by CPZ.
1,2-Benzodiazepine	It increases the antidepressant effect of CPZ.
Abametapir	It increases the CPZ serum levels.
Abemaciclib	Increased metabolism of abemaciclib may result when co-administered with CPZ.
Acetaminophen	Acetaminophen metabolism may slow down when co-administered with CPZ.
Amoxicillin	CPZ retards the excretion of amoxicillin.
Anakinra	CPZ metabolism increases.

**Table 3:** OZP, CPZ, CZP, and ARP mode of action and description.

Drug	Mode of action	Description
OZP	Blocks serotonin and DA receptors in the brain	A DA cum serotonin antagonist that is a new-generation medicine under APYs
CPZ	Blocks DA receptors	An old-generation medicine, which falls under typical antipsychotics
CZP	Blocks serotonin and DA receptors in the brain	A DA cum serotonin antagonist that is a new-generation medicine under APYs
ARP	It partially blocks DA receptors, and it also blocks serotonin receptors	Partial DA antagonist, serotonin antagonist, and a new-generation atypical antipsychotic drug

## Data Availability

Not applicable.
